# Use of antenatal care, maternity services, intermittent presumptive treatment and insecticide treated bed nets by pregnant women in Luwero district, Uganda

**DOI:** 10.1186/1475-2875-7-44

**Published:** 2008-03-01

**Authors:** Mpungu S Kiwuwa, Patrobas Mufubenga

**Affiliations:** 1 Clinical Epidemiology Unit, Faculty of Medicine, Makerere University, Uganda; 2 Ministry of Health Headquarters, National Malaria Control Programme, Uganda

## Abstract

**Background:**

To reduce the intolerable burden of malaria in pregnancy, the Ministry of Health in Uganda improved the antenatal care package by including a strong commitment to increase distribution of insecticide-treated nets (ITNs) and introduction of intermittent preventive treatment with sulphadoxine-pyrimethamine for pregnant women (IPTp-SP) as a national policy in 2000. This study assessed uptake of both ITNs and IPTp-SP by pregnant women as well as antenatal and maternity care use with the aim of optimizing their delivery.

**Methods:**

769 post-partum women were recruited from a rural area of central Uganda with perennial malaria transmission through a cross-sectional, community-based household survey in May 2005.

**Results:**

Of the 769 women interviewed, antenatal clinic (ANC) attendance was high (94.4%); 417 (57.7%) visiting initially during the 2^nd ^trimester, 242 (33.5%) during the 3^rd ^trimester and 266 (37.1%) reporting ≥ 4 ANC visits. About 537 (71%) and 272 (35.8%) received one or ≥ 2 IPTp-SP doses respectively. Only 85 (15.8%) received the first dose of IPTp-SP in the 3^rd ^trimester. ITNs were used by 239 (31.3%) of women during pregnancy and 314 (40.8%) delivered their most recent pregnancy outside a health facility. Post-partum women who lacked post-primary education were more likely not to have attended four or more ANC visits (odds ratio [OR] 3.3, 95% confidence interval [CI] 1.2–9.3).

**Conclusion:**

These findings illustrate the need to strengthen capacity of the district to further improve antenatal care and maternity services utilization and IPTp-SP uptake. More specific and effective community health strategies to improve effective ANC, maternity services utilization and IPTp-SP uptake in rural communities should be undertaken.

## Background

Uganda is one of the countries in sub-Saharan Africa with high maternal and neonatal morbidity and mortality rates. According to the Uganda Demographic Health Survey (UDHS) 2001, the maternal mortality ratio is estimated to be 504 per 100,000 live births, with a neonatal mortality rate of 40 per 1000, and these indicators have barely changed for the last two decades. Over 90% of the population live in highly endemic areas with perennial transmission while the 10% live in low transmission areas which are prone to malaria epidemics. Children are most vulnerable to malaria, with mortality rates being highest among children under five years of age. Malaria during pregnancy contributes to high maternal morbidity and mortality as a result of its contribution to severe anaemia, low birth weight, foetal loss and still births [1].

The threat of malaria in pregnancy to maternal and neonatal survival in Uganda is well-recognized by local communities in the country. Occult malaria in pregnancy (womb fever) is known by the local communities to be responsible for spontaneous abortions and foetal deaths. Consequently, these complications are believed to be responsible for some of the women's emotional stress, stigma, superstition, self-hatred, indulgence, divorce and ostracization in society [2]. One study in the country estimated the prevalence of malaria parasitaemia in pregnancy to be 62.1% [3], while severe maternal anaemia (Hb < 8 gdl^-1^) and low birth weight were found to be 18% and 12.4% respectively [2]. Sleeping under an insecticide-treated bed net (ITN) can reduce the risk of a pregnant woman being infected with malaria and reduce the risks of maternal anaemia and low birth weight [4]. Another preventive measure of intermittent preventive therapy with Sulfadoxine-pyrimethamine for malaria in pregnancy (IPTp-SP) in areas of high or seasonal transmission has been has been shown to increase both maternal haemoglobin levels and the infants' birth weight [5-8]. Sulphadoxine-pyrimethamine may act by treatment or prevention of new infections. The World Health Organization (WHO) recommends that all pregnant women in areas of stable malaria transmission should receive at least two doses of IPT after quickening (first noted movement of the foetus) during regularly/routinely scheduled antenatal clinic visits.

The Uganda Reproductive Health (RH) policy guidelines of 2000 recommend a schedule of a minimum four ANC visits [9], in which a complete antenatal care package can be delivered appropriately. According to this recommendation, a woman with an uncomplicated pregnancy is expected to make ANC visits; once in each of the first and second trimesters, and twice in the 3^rd ^trimester. The current antenatal care package includes: health education and counselling on pregnancy and emergency preparedness, nutrition, hygiene, birth plan, postpartum care, breast feeding, sexually-transmitted infection prevention and family planning. It also involves antenatal examinations, inter-current disease treatment, routine screening for syphilis, voluntary counselling and testing for HIV, prevention of mother to child transmission of HIV (PMTCT), periodic de-worming, nutrition supplementation and tetanus immunisations [10]. The National Malaria Control Programme priority intervention areas in 2001 included (IPTp-SP), vector control with emphasis on promoting large-scale use of insecticide-treated materials, indoor residual spraying and improved appropriate case management. Through this strategy the country expects to achieve a target of 75% pregnant women receiving complete IPTp-SP, 70% of households each with an insecticide treated net (ITN), and 100% clinical malaria cases receiving appropriate case management by the year 2010 [10]. It was observed that on average women make only two ANC visits during pregnancy and often at late gestational ages (>28 weeks) irrespective of parity, education or economic status [11], resulting in poor uptake of RH services by communities [12]. This is demonstrated by the fact that two years after implementation of the malaria in pregnancy control strategy, the two dose IPTp-SP coverage stood at only 5% in the central region despite efforts to improve these services (Quarterly PHC Monitoring Report January 2002). Pregnant women from rural areas are more likely to have placental malaria compared to women from urban areas [13], thus the need for more studies on antenatal care performance and IPTp-SP coverage in rural settings. A baseline community household survey was therefore conducted in rural central Uganda among women who had recently delivered to assess the use of antenatal care, maternal services, IPTp-SP and ITNs with the aim of improving uptake of an optimal antenatal care package.

## Methods

### Study setting

Luwero district in rural central Uganda was purposively selected for study because of its malaria endemicity, ethnic homogeneity and social stability. Luwero district borders Kiboga, Wakiso, Nakasongola and Mukono districts with a longitudinal location of 32°. It has a total area of 5,773.53 square km, with a total population of 474,627, and 24,681 annual expected pregnancies (National Census 2002). The district is sub-divided into 4 counties and 20 sub-counties. The national health policy (1999) established the health sub-district (HSD) as a functional subdivision, or service zone, of the district health system to bring quality essential care closer to the people. Within the HSD, exists a hierarchy of lower level health units. The leadership of the HSD is located in an existing hospital or a HC IV (Public or Private not for profit) located within the HSD. The HSDs in Luwero district include; Nakaseke, Katikamu North, Katikamu south, and Bamunanika. The district has 68 recognized health units able to offer antenatal care services. However, only 43% (59/136) of the parishes have at least one health unit, which shows limited access to health care. Currently ANC services are offered at all levels of care right from parish level (Health centre II) up to the national level (National and regional hospitals), complemented by private domiciliary clinics at village level. At public health units pregnant women get free ANC services. However ITNs are dispensed at subsidies ranging between 0 and 100%, as determined by local authorities, social marketing strategies or development partners.

Antenatal care services are offered by various categories of health care workers including private midwives and traditional birth attendants. SP is administered as directly observed therapy from the ANCs. The district has a hot and wet type of climate, with an average rainfall of 1,300 mm resulting into malaria transmission all year round (hyperendemic area). Malaria infections and morbidity have seasonal peaks after rains in March-May and in September-December. The district populace largely depends on agricultural earnings. Prior to the survey, the project team visited the study area and met the district local leaders and health team so as to establish dialogue and sensitize the community about the project. Permission to carry out the study was granted by Uganda National Council of Science and technology (UNCST) the national ethical board.

### Study design

A cross-sectional community survey of two health sub-districts of Luwero district namely Bamunanika and Kapeka, was conducted in May 2005. All post-natal women who had delivered their baby within the preceding five months were eligible for inclusion in the study. Bamunanika and Kapeka sub-counties were purposively selected based on similarity in population size, health unit number and annual expected pregnancy rates. The populations of Bamunanika and Kapeka were estimated at 24,848 and 24,068 respectively (Uganda population census 2002). It was estimated that a sample size of 384 mothers in each sub-county was required to calculate the true proportion of those using IPT within 5% points and assuming a 50% prevalence of at least four ANC visits. The research team comprised of six trained research assistants and two field supervisors. The study population was obtained using a multi stage sampling technique in which all parishes in the two chosen HSDs were sampled. In each HSD data was collected for a total duration of five days. With the help of field guides, the centre of each parish was identified and a bottle spinned to determine the initial direction where the sample was drawn. Households were consecutively visited and one eligible woman per household was interviewed until a pre-determined number of respondents per parish was obtained. Where more than one eligible woman was present in a household, a respondent among them was selected randomly.

### Data collection instruments

Participants were interviewed using a pre-tested semi structured questionnaire. A questionnaire was administered in the local language to each participant after obtaining informed consent. The questionnaire collected information about: a) Socio-demographic variables including age, religion, occupation, education level, distance to health units, marital status: b) antenatal care service utilization including number of antenatal care visits, health units visited, IPT intake and ITN use: c) maternity services including delivery process and place of delivery. Antenatal clinic cards were routinely requested from the participants to corroborate information provided by self report. Complete IPT uptake was defined as provision of at least two or more SP doses given at the ANC to women who were not sick [14]. The field supervisors regularly cross-checked on the completeness the data collected on a daily basis. Limitations of the study may include imprecise estimates of gestational ages and IPT intake during ANC visits due to potential recall bias and in some instances, lack of ANC cards to corroborate some of the information obtained by self report.

### Data analysis

Data was double entered into a computer using Epi info version 2000 and analysed using SPSS version 10.0. Continuous data was presented as means (standard deviation) SD and medians. Comparison of continuous data was done using the independent t-test and nonparametric tests (Mann-Whitney U test) where the assumptions of normality were unmet. Proportions were analysed using the Chi-square test or Fisher's exact test. Odds ratios and 95% confidence intervals (CI) were determined. Statistical significance was taken as P < 0.05.

## Results

### Socio-demographic characteristics of the study population

Seven hundred and sixty nine post-partum women participated in the study and their characteristics are shown in (Table 1). The mean age (standard deviation [SD]) of the participants was 24.7 (5.9) years. The group was predominantly made up of married women in the 20–29 years age-group 459 (59.7%); and 186 (24%) having attained post primary level of education. It is noted that there were high rates of unemployment amongst this study population 674 (87.6%); with only 31 (4%) engaged in subsistence farming. The majority of the mothers 625 (81.3%) had recently delivered within the previous four months. In terms of access to health care, the mean distance to the nearest ANC clinic was 3.9 km (SD) 3.2. Only 82 (10.7%) of the participants had been visited by a community health worker during their previous pregnancy.

**Table 1:** Socio-demographic characteristics of the participants (N = 769)*

**Characteristic**	n (%)
**Median age (quartiles), y**	24 (20–28)
**Parity**	
Para 1	136 (17.7)
Para > 1	632 (82.3)
**Marital status**	
Married	651 (84.7)
Single, separated or widowed	118 (15.3)
**Occupation status**	
Unemployed	674 (87.6)
Peasant	31 (4.0)
Trader	20 (2.6)
Casual laborer	6 (0.8)
Professional	19 (2.5)
Other	19 (2.5)
**Education status**	
None	79 (10.3)
Lower primary (P1–P4)	112 (14.6)
Higher primary (P5–P7)	390 (50.8)
Lower secondary (S1–S4)	164 (21.4)
Higher secondary (S5–S6)	6 (0.8)
Lower tertiary(certificate/diploma)	16 (2.1)
Distance to nearest health unit median (quartiles) Km	4 (1.6–5.6)
Period since delivery median (quartiles) mo	3 (1–4)

*Values are number percentage unless otherwise stated

### Antenatal care services utilization

Of the 769 participating women, 722 (94.4%) reported having visited an antenatal clinic during their most recent pregnancy (Table 2). Antenatal clinic attendance was verified among 344 (86%) participants who presented ANC records at the time of the interview. The mean number of self reported antenatal clinic visits during the recent pregnancy was 3.0 range (1–10). Antenatal clinic attendance did not differ among those who lived within a distance of 4 km from the nearest formal health unit compared to those living beyond, P = 0.84. Approximately 88% of the women made more than one ANC visit. The frequency of 4 or more self reported antenatal care visits among ANC attendees was 266 (37.1%). The majority of the women 417 (57.7%) reported attending the ANC for the first time in the second trimester, while 242 (33.5%) commenced in the third trimester. Women who had not attained post-primary education, 39 (90.7%), were almost three times more likely not to have visited an ANC compared to women who had completed primary education, 4 (2.2%); (OR 3.3, 95% CI 1.2–9.3). Of the ANCs visited by the women, the health centre III level was the most commonly attended locality. However, only 12 (1.6%) acknowledged visiting traditional birth attendants for antenatal care services.

**Table 2:** The use of Antenatal clinics, and IPT by pregnant women*

**Characteristic**	**n (%)**
No. with ANC card*	351 (46.0%)
**Number visiting ANC at least once**	722 (94.4%)
**No. visiting ANC at least twice**	676 (88.4%)
**Median No. of ANC visits (quartiles)**	3 (2–4)
**Timing of ANC visits**	
Median month of 1^st ^visit (quartiles)	5.7 (1.6)
Median month of 2^nd ^visit (quartiles)	6.8 (1.3)
Median month of 3^rd ^visit (quartiles)	7.7 (1.1)
Median month of 4^th ^visit (quartiles)	8.3 (1.0)
**Place of ANC attendance**	
TBA	12 (1.6%)
Private clinic	134 (18.2%)
HC II	101 (13.7%)
HC III	332 (45.2%)
HC IV	45 (6.1%)
Hospital	111 (15.2%)
**IPTp-SP**	
Took no IPTp-SP	215 (28.5%)
Took one dose of IPTp-SP	265 (35.3%)
Took more than one dose IPTp-SP	272 (36.2%)
**Gestational age 1^st ^IPTp-SP dose, mo†**	
<4	14 (2.6%)
4–7	438 (81.6%)
7.1–8.5	73 (13.6%)
>8.5	12 (2.2%)
**Gestational age 2^nd ^IPTp-SP dose, mo†**	
4–7	121 (45%)
8–8.5	108 (40%)
>8.5	40 (15%)

IPTp-SP indicates intermittent preventive treatment with sulphadoxine-pyrimethamine for malaria in pregnancy. *Sixteen women did not know if they received SP in the ANC.

† < 4 mo (1^st ^trimester), 4–7 mo (2^nd ^trimester), 7.1–8.5 mo (early 3^rd ^trimester), >8.5 (late 3^rd ^trimester)

### IPT uptake at the antenatal clinics

Approximately 544 (71.7%) of the postpartum women had taken at least one dose of SP while they were pregnant (Table 2). All SP treatments were presumptive (taken when they were not sick), and, they were all administered at an ANC. However fewer mothers 272 (35.8%) received at least two doses of IPTp-SP at an ANC visit. Only 417 (57.7%) reported taking the first dose of IPTp-SP within the second trimester of pregnancy (4–7 mo) and occasionally in 9% of women, it was given during the first trimester (<4 mo). Consumption of other preventive anti-malarials through mechanisms other than IPTp-SP was uncommon (1.5%) in this study population. More primigravidae 52 (19.1%) took two or more doses of IPTp-SP compared to multigravidae 47 (17.8%), however the difference did not reach statistical significance, P = 0.69. Lack of post primary education was associated with failure to use at least one dose of IPTp-SP; OR 2.2, 95%CI (1.4–3.3).

### Bed net use

Only 239 (31.3%) of women stated that they used a bed net during their previous pregnancy, and only 116 (48.5%) reported a net re-treatment with an insecticide within the last 6 months. Most nets were obtained from the commercial sector with no long-lasting impregnated nets (LLINs) available on the market. The mean gestational age at inception of net use was 2.5 months. Women experiencing pregnancy for the first time, 47 (34.6%), were more likely to report using a bed net compared to the multigravidae 191 (30.5%); however this difference did not reach statistical significance, P = 0.356.

### Maternity services

Most women delivered in health centers (28.7%); 193 (26.4%) delivered from home, 133 (18.2%) in private maternity homes and 101 (13.8%) in a hospital (Table 3). Approximately 455 (59.2%) women gave birth with a skilled attendant present. Others delivered either by themselves or with the help of relatives, friends or traditional birth attendants.

**Table 3:** Maternity services utilization*

**Variable**	n (%)
Gestation age at delivery median (quartiles), mo	9 (9–9)
**Nature of birth attendant**	
Self	35 (4.6%)
Relative/friends	126 (16.4%)
Traditional Birth attendant	153 (19.9%)
Private midwife/trained health worker	139 (18.1%)
Public Health Unit attendant	316 (41.1%)
**Place of delivery**	
Home	193 (26.4%)
TBA premises	94 (12.9%)
Maternity home	133 (18.2)
HC II	53 (7.3)
HC III	130 (17.8)
HC IV	26 (3.6)
Hospital	101 (13.8)

*Values are number percentage unless otherwise stated.

HC indicates Health centre

## Discussion

The study indicates a high rate of antenatal care attendance as similarly observed in other African countries with IPTp-SP policy implementation [15] and offers the potential for implementing the nationally recommended approaches to the prevention and control of malaria. Six years after the introduction of IPT in Uganda, only a small percentage of women in this rural area are benefiting from this policy. More than two thirds of women attending antenatal care and delivering from formal health units received at least one dose, but fewer than 40% received the recommended full two dose SP regimen. These findings are similar to studies done else where in Africa, where six years after the introduction of this policy, a hospital-based study in urban Kisumu, Kenya reported (43.4%) receiving one dose of SP and (23.7%) receiving two doses [13]; a hospital-based study in urban Malawi reported 45.1% receiving one dose and 30.6% receiving two doses [6]; a community-based study in rural Malawi reported 75.7% receiving one dose and 43.7% receiving two or more doses [16]; and a nation wide survey reported 67.5% receiving one dose and 29.3% receiving two or more doses (National Statistical Office Malawi 2001).

It is noteworthy that ANC cards may not be routinely available after a woman has delivered, suggesting that the reported use of malaria preventive measures in our study might be an overestimate. In this rural setting, the coverage of pregnant women with the recommended malaria preventive measures (ITNs and IPT) in 2005 was <5% of the targets set by Roll Back Malaria movement for 2005. Only 30% of women slept under a bed net and only 36.2% received two or more doses of SP as presumptive treatment, and these tended to be mutually exclusive with only 13% of women being covered by both these malaria preventive measures. Primigravidae were more likely to sleep under a net compared to multigravidae, although this finding has not been consistent in studies done elsewhere [17-19]. The community perceptions regarding malaria as an important complication among vulnerable groups such as primigravidae may possibly explain this observation. Previous studies in Uganda have revealed that perceiving malaria as an important complication in pregnancy appears to be an important factor in motivating pregnant women to participate in a program [3,20]. The majority of the mothers reported receiving antenatal care from formal health units, however approximately 60% delivered their most recent pregnancy outside a formal health facility. Similarly in Hoima district of western Uganda, approximately 60% of women delivered outside a health facility [21]. Other studies have reported 55% of women delivering from home [3], only 58% delivering from hospital in Malawi [7], 51% delivering from a health institution and 49% had delivered under TBA care in Nigeria [22]. The possible reasons for low deliveries at formal health units could be related to costs, cultural factors, health worker's attitudes towards pregnant women and the perceived quality of care at the health units [3,21,23]. Given the projected absence of adequate cadres of skilled birth attendants in many countries for years to come calls for innovative approaches to improve perinatal health in this community. The newly initiated scaling-up of provision of free LLINs to vulnerable groups countrywide is part of Ugandan commitment to improve access to ITNs for pregnant women as a whole. It is hoped that through community health initiatives such as health education and promotion, some of the socio-economic inequalities that hinder access of rural communities to healthcare service utilisation will be addressed.

## Conclusion

In conclusion, this baseline survey confirms that ANC attendance and delivery of a full IPT regimen are sub-optimal. This results in limited access to information on malaria prevention in pregnancy, such as the benefits of IPT and ITNs. Given findings of this baseline study and others to come in Uganda it is important to identify strategies to increase IPTp-SP uptake in rural areas. Optimising IPT uptake during ANC services would be the most rational option given high attendance rates of the formal health sector. To this end, the malaria control programme of the Ministry of Health, Uganda, designed a programme to strengthen the capacity of the district for improved ANC performance and hence IPT uptake. Through the use of trained community owned resource persons, pregnant women are periodically made aware about the consequences of malaria in pregnancy and the necessity of an early ANC visit so as to benefit from administration of a full IPT regimen. The implementation of such a strategy is subject to further evaluation.

## Authors' contributions

PM conceived of the study, participated in it's design, coordination of the data collection process and draft of the manuscript. MSK participated in the design and coordination of the study, performed the statistical analysis and drafted the manuscript. All authors read and approved the final manuscript.

## References

[B1] Steketee RW, Nahlen BL, Parise ME, Menendez C (2001). The burden of malaria in pregnancy in malaria-endemic areas. Am J Trop Med Hyg.

[B2] Mufubenga P (2001). The burden of Malaria in pregnancy and the prospects of integrating its activities into the reproductive health services in Uganda.

[B3] Ndyomugyenyi R, Neema S, Magnussen P (1998). The use of formal and informal services for antenatal care and malaria treatment in rural Uganda. Health Policy Plan.

[B4] Hawley WA, ter Kuile FO, Steketee RS, Nahlen BL, Terlouw DJ, Gimnig JE, Shi YP, Vulule JM, Alaii JA, Hightower AW, Kolczak MS, Kariuki SK, Phillips-Howard PA (2003). Implications of the western Kenya permethrin-treated bed net study for policy, program implementation, and future research. Am J Trop Med Hyg.

[B5] Parise ME, Ayisi JG, Nahlen BL, Schultz LJ, Roberts JM, Misore A, Muga R, Oloo AJ, Steketee RW (1998). Efficacy of sulfadoxine-pyrimethamine for prevention of placental malaria in an area of Kenya with a high prevalence of malaria and human immunodeficiency virus infection. Am J Trop Med Hyg.

[B6] Rogerson SJ, Chaluluka E, Kanjala M, Mkundika P, Mhango C, Molyneux ME (2000). Intermittent sulfadoxine-pyrimethamine in pregnancy: effectiveness against malaria morbidity in Blantyre, Malawi, in 1997–99. Trans R Soc Trop Med Hyg.

[B7] Schultz LJ, Steketee RW, Chitsulo L, Macheso A, Kazembe P, Wirima JJ (1996). Evaluation of maternal practices, efficacy, and cost-effectiveness of alternative antimalarial regimens for use in pregnancy: chloroquine and sulfadoxine-pyrimethamine. Am J Trop Med Hyg.

[B8] Shulman CE, Dorman EK, Cutts F, Kawuondo K, Bulmer JN, Peshu N, Marsh K (1999). Intermittent sulphadoxine-pyrimethamine to prevent severe anaemia secondary to malaria in pregnancy: a randomised placebo-controlled trial. Lancet.

[B9] WHO (2000). Expert committee on malaria Twentieth report WHO document.

[B10] Ministry of Health Uganda (2005). Health Sector Strategic Plan II, 2005/06 –   2009/2010. 1; 2009/2010.

[B11] PREMA-EU (2003). The Newsletter of the Pregnancy Malaria Anaemia Network. PREMA-EU Newsletter.

[B12] Ministry of Health Uganda (2001). Annual Health Sector Performance report, financial year 2000/2001.

[B13] van Eijk AM, Ayisi JG, ter Kuile FO, Otieno JA, Misore AO, Odondi JO, Rosen DH, Kager PA, Steketee RW, Nahlen BL (2004). Effectiveness of intermittent preventive treatment with sulphadoxine-pyrimethamine for control of malaria in pregnancy in western Kenya: a hospital-based study. Trop Med Int Health.

[B14] Guyatt HL, Noor AM, Ochola SA, Snow RW (2004). Use of intermittent presumptive treatment and insecticide treated bed nets by pregnant women in four Kenyan districts. Trop Med Int Health.

[B15] Hill J, Kazembe P (2006). Reaching the Abuja target for intermittent preventive treatment of malaria in pregnancy in African women: a review of progress and operational challenges. Trop Med Int Health.

[B16] Holtz TH, Kachur SP, Roberts JM, Marum LH, Mkandala C, Chizani N, Macheso A, Parise ME (2004). Use of antenatal care services and intermittent preventive treatment for malaria among pregnant women in Blantyre District, Malawi. Trop Med Int Health.

[B17] Marchant T, Schellenberg JA, Edgar T, Nathan R, Abdulla S, Mukasa O, Mponda H, Lengeler C (2002). Socially marketed insecticide-treated nets improve malaria and anaemia in pregnancy in southern Tanzania. Trop Med Int Health.

[B18] ter Kuile FO, Terlouw DJ, Phillips-Howard PA, Hawley WA, Friedman JF, Kariuki SK, Shi YP, Kolczak MS, Lal AA, Vulule JM, Nahlen BL (2003). Reduction of malaria during pregnancy by permethrin-treated bed nets in an area of intense perennial malaria transmission in western Kenya. Am J Trop Med Hyg.

[B19] van Eijk AM, Ayisi JG, ter Kuile FO, Slutsker L, Otieno JA, Misore AO, Odondi JO, Rosen DH, Kager PA, Steketee RW, Nahlen BL (2004). Implementation of intermittent preventive treatment with sulphadoxine-pyrimethamine for control of malaria in pregnancy in Kisumu, western Kenya. Trop Med Int Health.

[B20] Mbonye AK, Neema S, Magnussen P (2006). Perceptions on use of sulfadoxine-pyrimethamine in pregnancy and the policy implications for malaria control in Uganda. Health Policy.

[B21] Kyomuhendo GB (2003). Low use of rural maternity services in Uganda: impact of women's status, traditional beliefs and limited resources. Reprod Health Matters.

[B22] Nwakoby BN (1994). Use of obstetric services in rural Nigeria. Journal of the Royal Society of Health.

[B23] Lalonde AB, Okong P, Mugasa A, Perron L (2003). The FIGO Save the Mothers Initiative: the Uganda-Canada collaboration. Int J Gynaecol Obstet.

